# Modulation of physical exercise intensity in motor‐cognitive training of adults using the SKILLCOURT technology

**DOI:** 10.14814/phy2.70136

**Published:** 2024-12-05

**Authors:** Gülsa Erdogan, Bettina Karsten, Lutz Vogt, Andreas Mierau, Thorben Hülsdünker

**Affiliations:** ^1^ Department of Sports Medicine and Exercise Physiology Goethe University Frankfurt Frankfurt am Main Germany; ^2^ European University of Applied Sciences (EU|FH) Berlin Germany; ^3^ Department of Exercise and Sport Science LUNEX Differdange Luxembourg; ^4^ Luxembourg Health & Sport Sciences Research Institute (LHSSRI) Differdange Luxembourg

**Keywords:** brain training, exergaming, heart rate, high‐intensity‐training, oxygen uptake

## Abstract

Motor‐cognitive training and exergaming often only reach low‐to‐medium intensities that limits their training efficiency. This study evaluated the physiological profile of different exercises on a novel motor‐cognitive training technology designed to cover a broad range of exercise intensities. Twenty‐six healthy trained adults (17 males, 23.7 ± 3.8 years) performed five motor‐cognitive training tasks on the SKILLCOURT technology. Oxygen consumption (VO_2_), heart rate (HR), blood [lactate], perceived physical exertion (RPE) responses, and metabolic equivalent (MET) were assessed and compared to an incremental treadmill ramp test determining the maximal oxygen consumption (VO_2max_) and maximal heart rate (HR_max_). Computer‐based cognitive training served as control condition. Motor‐cognitive exercises reached a higher %VO_2max_ and %HR_max_ levels when compared to computer‐based training (*p* < 0.001). Average intensity varied significantly between motor‐cognitive tasks, with %VO_2max_ ranging from 22% to 81% (*p* < 0.001), %HR_max_ from 49% to 89% (*p* < 0.001), METs from 3.57 to 13.37 (*p* < 0.001), blood [lactate] from 0.93 to 7.81 mmol·L^−1^ (*p* < 0.001), and RPE from 8.5 to 16.4 (*p* < 0.001). Motor‐cognitive training covers a wide range of exercise intensities. This supports individual training subscription and allows high‐intensity training to facilitate cardio‐vascular adaptations and neural plasticity.

## 1 INTRODUCTION

Physical activity (PA) plays an important role not only for supporting cardiovascular but also cognitive performance and health. Especially when combined with cognitive tasks it triggers neurogenesis, synaptogenesis, and neural protection throughout the adult lifespan (Kempermann et al., 2010; Raichlen & Alexander, 2017). Research has shown that incorporating cognitive tasks into PA can be more effective for cognition than cognitive and physical training alone (Herold et al., 2018; Torre & Temprado, 2022). In this context, exergaming, physical‐cognitive training, and motor‐cognitive (m‐c) training have been developed to complement the synergistic effects of physical and cognitive exercise.

For both, neurophysiological and cardiovascular adaptations, the intensity of physical exercise is of particular importance. Exercise triggers the release of neurotrophic factors like the brain‐derived neurotrophic factor (BDNF) that support synaptic transmission, plasticity, growth, and survival (Lu et al., 2013). However, BDNF concentrations depend on exercise intensity and are particularly elevated following high‐intensity interval exercises (Reycraft et al., 2020). Similarly, cardiovascular and metabolic adaptations are enhanced following high‐intensity when compared to moderate intensity training (Helgerud et al., 2007; Milanović et al., 2015). This does not only apply to trained athletes but has also been confirmed for elderly and patient populations (Marriott et al., 2021).

To exploit the full potential of m‐c training and exergaming for supporting neural and cardiovascular health and performance, exercises must cover a wide range of intensities which particularly includes high intensity physical exercise. There are different classifications of high intensity exercise that vary between >72% (Høydal, 2017) and >90% (Vaccari et al., 2020) maximal oxygen consumption (VO_2max_), which, according to the ACSM guidelines, corresponds to vigorous activity (64%–90% of VO_2max_) (Garber et al., 2011). Pratt et al. (2013) reported an average of 84% VO_2max_ during an interval running protocol of 5 × 2 min at VO_2max_ intensity. Similar intensities of 83% and 80%–84% VO_2max_ have been reported by Rozenek et al. (2016) and Vaccari et al. (2020), respectively, both using a high‐intensity cycling protocol. These findings suggest that high‐intensity exercises in exergaming or m‐c training should reach an average %VO_2max_ of at least 80%. According to the ACSM guidelines for vigorous intensity, this should be accompanied by 77%–95% of maximal heart rate (HR_max_), a Rate of Perceived Exertion (RPE) rating between 14–17 and 6–8.7 in metabolic equivalents (MET) (Garber et al., 2011). In terms of blood [lactate] >6 mmol/L have been observed following a 4 × 90 s running protocol (90 s break) (Warr‐di Piero et al., 2018).

While high intensity is important during training, recent reviews of m‐c training and exergames reported exercise typically ranging between light and vigorous intensities (Davis et al., 2022; Marshall & Linehan, 2021; Mohd Jai et al., 2021). Specifically, Davis et al. (2022) reported that only eight out of 35 exergames reached a vigorous exercise intensity. The intensity classification was often based on MET or age‐predicted %HR_max_ which limits a comprehensive classification of the intensity profile. Especially MET's have been criticized for their poor relation to established relative parameters such as %HR_max_ or %VO_2max_ (Warner et al., 2022). Further, as VO_2max_ and other physiological variables like lactate are often not considered (Aygün & Çakir‐Atabek, 2021; Aygün & Çakır‐Atabek, 2023; Graves et al., 2010; Perusek et al., 2014; Röglin et al., 2021) this limits the options for individual training prescription. To the best of our knowledge, only the ExerCube, which is a physically immersive exergame that adapts to the player's physical performance considers a greater range of intensity by adapting the game to the individual physical effort (Martin‐Niedecken et al., 2020). For an ExerCube training session with five intervals and an overall duration of 25 min, Ketelhut et al. (2021) reports average %HR_max_ and %VO_2max_ of 86% and 66%, respectively. However, the ExerCube training intensity of 66%VO_2max_ is still comparatively low and below the typical 80%VO_2max_ during HIT (Pratt et al., 2013; Rozenek et al., 2016; Vaccari et al., 2020).

Together, current m‐c trainings and exergames are limited by mostly low‐to‐moderate exercise intensities not reaching high physical exercise intensity (>80% VO_2max_) as well as a limited set of investigated physiological parameters. The SKILLCOURT technology that has been specifically designed to provide a wide range of physical and cognitive demands in different types of m‐c trainings. This approach is based on the guided plasticity facilitation model (Herold et al., 2018) and it attempts to leverage the synergistic effects of cognitive and physical effort. In this context, reaching high exercise intensities is of particular importance as it supports cardio‐vascular adaptations not only in athletes but also elderly participants (Engel et al., 2018; Liu et al., 2024; Marriott et al., 2021; Rebsamen et al., 2019). Further, it increases the release of neurotrophic factors such as BDNF (Reycraft et al., 2020) that is suggested a key element to support neuroplastic changes in motor‐cognitive and physical cognitive training (Herold et al., 2018; Raichlen & Alexander, 2017). The SKILLCOURT has previously been shown as valid and reliable for a number of m‐c testing (Friebe et al., 2023; Hülsdünker et al., 2023) and it provides a better transfer to football relevant performance when compared to change‐of‐direction training (Friebe et al., 2024). Exercises are performed either in a stationary position by tapping with the left and right foot or they are dynamic by running to designated target fields. It may be reasonable to assume that due to the court dimension (up to 5 × 5 m), running distances up to 150 m per exercise as well as rapid changes of direction, the SKILLCOURT can cover both low (stationary) and high (running/sprinting) exercise intensities. However, it remains to be evaluated if m‐c exercises can reach intensities equivalent to high‐intensity training, as cognitive tasks interfere with the motor component (i.e., motor‐cognitive interference) and increased cognitive demands affect agility performance (Büchel et al., 2022) and probably physiological load. Accordingly, dual‐task costs arising from the combination of cognitive and motor tasks may limit the maximum exercise intensity when compared to physical training alone.

This study evaluated oxygen uptake (VO_2_; ml/kg/min), blood [lactate; mmol/l^−1^], HR (b.min^−1^), metabolic equivalent (MET), and RPE during m‐c exercises on the SKILLCOURT. Intensity was compared between motor‐cognitive exercises performed on the SKILLCOURT as well as between motor‐cognitive and computer‐based cognitive tasks. It was hypothesized that different m‐c tasks on the SKILLCOURT cover a wide range of exercise intensities, including high‐intensity exercise. Further, m‐c tasks should exceed physiological demands of computer‐based exercises independent of the task.

## 2 MATERIALS AND METHODS

### 2.1 Sample size calculation

Based on results by Çakir‐Atabek et al. (2019), an a priori sample size calculation was conducted using G*Power (version 3.1.9.6, University of Kiel, Germany) (Faul et al., 2007). Based on an effect size for differences in oxygen uptake between a dancing and fighting video game exercise of d = 1.5 (Çakir‐Atabek et al., 2019) converted into partial eta square (η_p_^2^) for ANOVA analysis revealed five participants for an alpha level of 0.05 and a test power of 0.9. However, in this study, differences between motor‐cognitive exercises were considered lower due to involving two stationary exercises varying in task demands (fixed interstimulus interval versus adaptive) and two all‐out agility tasks differing in cognitive load. Therefore, the effect size was expected to be considerably lower and the required sample size was calculated based on a medium effect size of η_p_^2^ = 0.1 resulting in a required sample size of minimum 16 participants. With 26 athletes included in the final analysis this study was considered sufficiently powered.

### 2.2 Participants and ethics

Twenty‐eight healthy adults (17 males, 9 females; 23.7 ± 3.8 years; VO_2max_: 54.5 ± 7.9 mL·kg^−1^·min^−1^) participated in this study. Based on their VO_2max_ categorized as good to superior and based on age and gender participants were considered as trained (Gibson et al., 2019) and corresponded to Tier 2 (Trained/Developmental) of the participant classification framework proposed by McKay et al. (2022). Exclusion criteria comprised orthopedic injuries, chronic diseases, or cognitive disabilities. Participants were requested not to engage in physically strenuous activities and to maintain their dietary habits for the duration of the study. Participants had to refrain from consuming alcohol or exceeding their daily caffeine intake on both test days of the study and they were asked to refrain from food intake 3 h before data acquisition. All participants were informed about the study's objective, experimental procedures, and associated risks, and written consent was obtained. The study was approved by the Luxembourgish national research ethics committee (CNER) in accordance with the Declaration of Helsinki (202207/01 v2.0).

### 2.3 Experimental protocol

**FIGURE 1:**
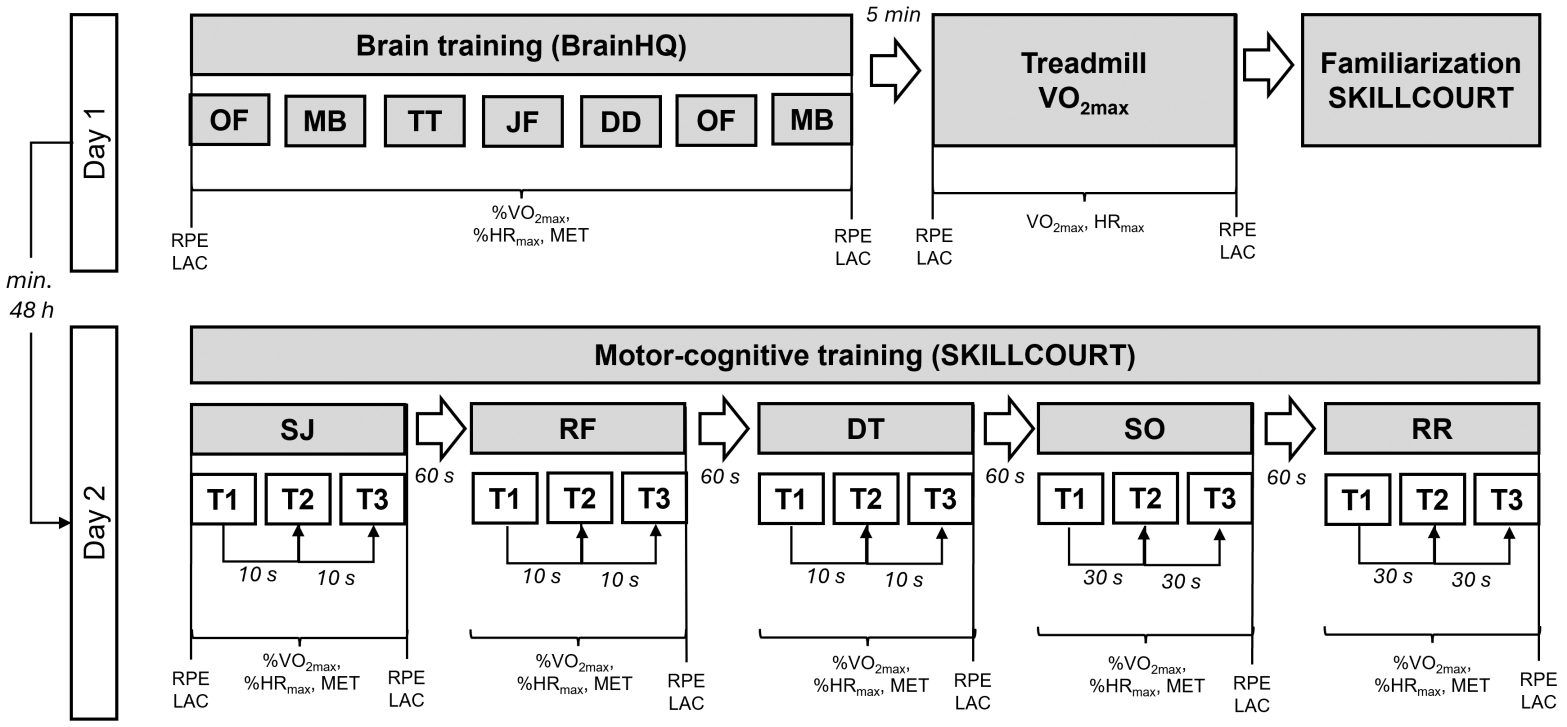
Overview of the experimental protocol with Treadmill VO_2max_ and computer‐based cognitive training on day 1 and motor‐cognitive training on the SKILLCOURT on day 2. Both, the computer‐based training and motor‐cognitive session had a duration of 20–25 min. RPE = rate of perceived exertion, LAC = lactate, MET = metabolic equivalents, T1‐T3 = trial 1–3 performed for each motor‐cognitive exercise. Tasks during the computer‐based cognitive training: OF = optic flow, MB = mind bender, TT = target tracker, JF = juggle factor, DD = double decision. Tasks during the motor‐cognitive session on the SKILLCOURT: SJ = Shape Jump, RF = Remember Forms, DT = Dual Task, SO=Sorting, RR = Random Run Plus.

The experimental protocol is illustrated in Figure 1. Participants visited the laboratory on 2 days separated by at least 48 h. To avoid the effects of circadian rhythm, test times during the days differed by a maximum 2 h. On day 1, participants completed a computer‐based cognitive training (BrainHQ, Posit Science, San Francisco, CA, USA) on a laptop. After a 5 min recovery period, this was followed by a treadmill ramp test to determine VO_2_max and HR_max_. Afterwards participants were familiarized with the five m‐c SKILLCOURT exercises. Participants performed the m‐c exercises on test day two. Both, the cognitive and m‐c sessions had a duration of 20–25 min. The computer‐based training was performed during the treadmill session that included familiarization with the SKILLCOURT technology. Accordingly, the treadmill and computer‐based training must run before the motor‐cognitive training. There was no carry‐over effect expected between cognitive and motor‐cognitive training in both directions as tasks were performed on separate test days (min 48 h), and there is no practice or learning effect between motor‐cognitive and cognitive training.

During all assessments (cognitive training, ramp test, SKILLCOURT test), the participant's gas exchange was measured continuously breath by breath using a MetaMax 3B analyzer (CORTEX Biophysik GmbH, Leipzig, Germany). HR was measured continuously using a Polar H7 sensor (Polar Elektro, Kempele, Finland). Participants rated their rate of perceived exertion (RPE; 6 to 20) before and after each session and after each SKILLCOURT exercise (Borg, 1970). Earlobe blood lactate samples were collected before and immediately after the cognitive training, the ramp test and after each SKILLCOURT exercise and analyzed using a Biosen C‐Line lactate analyzer (EKF‐diagnostic GmbH, Barleben, Germany).

### 2.4 Treadmill ramp test (VO_2_max)

The incremental ramp‐like test to exhaustion was conducted on a treadmill (h/p/cosmos®, Pulsar®, Nussdorf, Germany). Two treadmill protocols were used, one starting at 6 km·h^−1^ and the other at 8 km·h^−1^, at 1% incline (Jones & Doust, 1996). Participants who exercised less than three times per week started the protocol at 6 km·h^−1^, whereas those who exercised more than three times per week started at 8 km·h^−1^ to account for expected differences in exercise capacity. Following a 3‐min warm‐up at the starting velocity, the treadmill speed was increased by 1 km·h^−1^ every 30 s. After reaching 16 km·h^−1^, the velocity remained constant, and inclination was increased by 1% per min (Born et al., 2017). Participants were verbally encouraged throughout the test. When participants reached exhaustion, the test was terminated. The treadmill protocol lasted between 8 and 12 min (Midgley et al., 2008; Thompson et al., 2010). A valid VO_2max_ was assumed when a leveling off in VO_2_ (<150 mL·min^−1^) occurred despite an increase in treadmill speed or inclination. In case of no VO_2_ plateau, secondary criteria such as RER ≥1.1, blood [lactate] ≥8 mmol·L^−1^, and RPE ≥18 were used as indicators of maximal effort indicating a peak oxygen uptake (VO_2peak_) (Thompson et al., 2010).

### 2.5 Cognitive exercises (brain HQ)

The computer‐based cognitive session included five exercises from BrainHQ, targeting different cognitive functions. Each exercise was performed once, except for Optic Flow and Mind Bender, which were conducted twice to ensure a similar duration as for the SKILLCOURT session. The task was explained to the participants before the start of the training. During training the difficulty was continuously adjusted based on the participant's performance by the BrainHQ software.

*Optic Flow* aims to train visual attention and visual processing speed. Participants had to identify objects in a dynamic visual scene that matched a target object. *Mind Bender* focusses on the participants' cognitive flexibility. Participants had to switch between different stimulus–response rules and provide their answers as fast as possible while avoiding errors. *Target Tracker* is designed to address divided attention. Participants were presented with targets to be tracked among moving distractors. After the end of each trial the tracked objects had to be identified. *Juggle Factor* trains short‐term and working memory. A sequence of numbers displayed into moving circles had to be memorized and recalled in correct order and location. *Double Decision* requires quick processing of visual information which addresses the useful field of view. Participants had to identify the orientation and location of two objects (car and road sign) that were briefly presented in the center (car) and periphery (road sign) of the visual field.

### 2.6 Motor‐cognitive exercises (SKILLCOURT)

**FIGURE 2:**
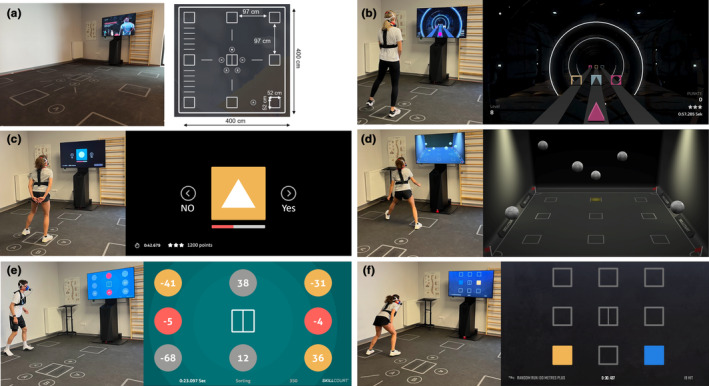
(a) Overview of the setup and dimensions of the SKILLCOURT technology. (b) Illustration of the *Shape Jump* exercise. (c) Illustration of the *Remember Forms* exercise. (d) Illustration of the *Dual Task* exercise. (e) Illustration of the *Sorting* exercise. (f) Illustration of the *Random Run Plus* exercise. Participants shown in the figure confirmed the use of the images for publication purposes.

The Skillcourt setup and motor‐cognitive exercises are presented in Figure 2. Five exercises were performed on a 4 × 4 m SKILLCOURT field (Figure 2a). All exercises followed the combination of motor and cognitive components into motor‐cognitive (m‐c) exercises. Based on preliminary tests, exercises were considered low, medium, and high intensity according to their physiological demands. The sequence of exercises was fixed and run from lowest to highest intensity as determined in the preliminary tests. This minimized variability in carry over effects which would have been substantial with counterbalancing or randomization designs. Each exercise consisted of three trials. For low‐ and medium intensity trainings, a 10 s break was integrated between trials. High‐intensity exercises had a break of 30 s. This setup was chosen to resemble realistic training conditions in m‐c training. Participants were verbally motivated to perform at their best during the session.

*Shape Jump* focusses on decision making and conflict inhibition. Participants had to match a colored (red, blue, or yellow) object (ball, square, or triangle) to a gate on one of three lanes with the same shape while ignoring the color. Participants were instructed to react as fast as possible while avoiding errors. Lanes were selected by stepping to the left or right response fields on the SKILLCOURT (Figure 2b). Each trial lasted 1 min. *Remember Forms* addresses working memory and decision making. A sequence of images containing a symbol on a colored background was presented. Participants had to decide if the image presented matched the one presented one trial before (1‐back) or not, by stepping on the corresponding response field on the SKILLCOURT (Figure 2c). Each trial lasted for 1 min and participants had to react as fast as possible while avoiding errors. *Dual Task* addressed the participant's ability to track multiple objects while performing a reactive agility task. Participants had to track two out of five balls moving on the screen (multiple object tracking) while at the same time running to highlighted target fields on the SKILLCOURT (reactive agility). Three rounds of 10 s were performed per trial (Figure 2d). Participants were instructed to reach as many fields as possible while not losing track of the balls. In the *Sorting* exercise, each outer target field of the SKILLCOURT is assigned to a number. Participants had to run to the fields in ascending order as fast as possible. After all eight outer fields were triggered in the correct order, all fields were assigned a new number. One trial last for 1 min where participants had to reach as many fields as possible while avoiding errors (Figure 2e). In the *Random Run* (*Plus*) *100 m* one of the outer target fields is highlighted in yellow indicating the next target for the run. A second field is highlighted in blue indicating the following target field. In the cognitive domain, this task requires planning ability and to a certain degree working memory. Target field sequence is randomized, and the system calculates the distance until 100 m is reached. Participants were instructed to finish the run as quickly as possible (Figure 2f).

Exercises on the SKILLCOURT included two stationary tasks with stepping movements (*Shape Jump*, *Remember Forms*) one task with running movements (*Dual Task*) and two tasks requiring sprints (*Sorting, Random Run Plus*).

### 2.7 Data analysis

Two participants were excluded for failing to meet the criteria for VO_2max_ or VO_2peak_. VO_2max_ was determined as the highest VO_2_ in a 30 s moving average during the incremental ramp test. The same applied to the definition of the maximum heart rate (HR_max_). VO_2_ during brain training was expressed as the average VO_2_ over the duration of the training period. For the SKILLCOURT, VO_2_ was calculated for each training by averaging VO_2_ during the three trials. For both the cognitive and m‐c exercises, 5‐s intervals were used. Similarly, HR was determined as the average HR during the cognitive and m‐c tasks. VO_2_ and HR values obtained in cognitive training and SKILLCOURT training were expressed as percentage VO_2max_ (%VO_2max_) and percentage HR_max_ (%HR_max_) relative to the ramp test. Metabolic equivalent (MET) was calculated considering age, gender, weight, and height by using the Harris‐Benedict Equations indicated in (1) and (2) (Harris & Benedict, 1918).

(1) $$\begin{array}{c} \;\mathrm{RMR}\;\text{Male}\;\left(\text{kilocalories}\;\mathrm{per}\;\mathrm{day}\right)\\ \;=66.4730+5.0033\;\left(\text{Height};\mathrm{cm}\right)\\ \;+13.7516\;\left(\text{Weight};\mathrm{kg}\right)-6.7550\;\left(\mathrm{Age};\mathrm{yrs}\right) \end{array}$$

(2) $$\begin{array}{c} \mathrm{RMR}\;\text{Female}\;\left(\text{kilocalories}\;\mathrm{per}\;\mathrm{day}\right)\\ \;=655.0955+1.8496\;\left(\text{Height};\mathrm{cm}\right)\\ \;+9.5634\;\left(\text{Weight};\mathrm{kg}\right)-4.6756\;\left(\mathrm{Age};\mathrm{yrs}\right) \end{array}$$

To convert the results of Equations 1 and 2 to mL·kg^−1^·min^−1^, Equation 3 (Ainsworth et al., 2011) was used:

(3) $$\begin{array}{c} \frac{\text{kcal}\cdot {\mathrm{day}}^{-1}}{1440}=\text{kcal}\cdot \min^{-1};\frac{\text{kcal}\cdot \min^{-1}}{5}\\ =L\cdot \min^{-1};\frac{L\cdot \min^{-1}\;}{\left(\text{weight};\mathrm{kg}\right)\;x\;1000}=\mathrm{mL}\cdot {\mathrm{kg}}^{-1}\cdot \min^{-1} \end{array}$$

The result of Equation 3 was implemented in Equation 4 to calculate the individual MET value:

(4) $$\;\mathrm{MET}\;\text{value}=\frac{VO_{2}\;\left(\mathrm{mL}\cdot {\mathrm{kg}}^{-1}\cdot \min^{-1}\right)}{\text{Harris}-\text{Benedict}\;\mathrm{RMR}\;\left(\mathrm{mL}\cdot {\mathrm{kg}}^{-1}\cdot \min^{-1}\right)}$$

In addition to each individual exercise, the average exercise intensity of the m‐c training was calculated across tasks. This indicator of the training session intensity allowed a better comparison to the computer‐based training and provides a comparison of physiological demands between a typical cognitive and motor‐cognitive training session. As tasks on the SKILLCOURT were varying in duration, a weighted average based on exercise duration was used for all physiological intensity variables (%VO_2max_, %HR_max_, MET, and RPE).

The %VO_2max_, %HR_max_, MET, and RPE of the cognitive training, all individual m‐c exercises and the m‐c session as a whole were then classified according to the ACSM exercise intensity categories (Garber et al., 2011).

### 2.8 Statistical analysis

The Shapiro–Wilk test revealed normal distribution in all, except five out of 35 variables. Despite these minor violations of normal distribution, a repeated measures analysis of variance (ANOVA) was considered most appropriate based on to the well‐established robustness of the ANOVA to violations of the normal distribution assumption for larger samples (Pagano, 2013).

To determine differences in average intensity between cognitive and m‐c (SKILLCOURT) training, paired *t*‐tests were applied to all variables (VO_2_, HR, MET, blood [lactate], and RPE). Differences in exercise intensity between m‐c trainings tasks on the SKILLCOURT were determined using repeated measures ANOVA with the factor “condition” (Shape Jump, Remember Forms, Dual Task, Sorting, and Random Run Plus). ANOVA analyses were performed for %VO_2max_, % HR_max_, MET, blood [lactate], and RPE. The Mauchly test assessed sphericity, and the Greenhouse–Geisser correction was applied if sphericity was violated. Bonferroni correction was used for post‐hoc tests. Effect sizes were considered small (d > 0.2, η_p_^2^ > 0.01), medium (d > 0.5, η_p_^2^ > 0.06), or large (d > 0.8, η_p_^2^ > 0.14). The significance level was set at *p* < 0.05.

## 3 RESULTS

### 3.1 Comparison between cognitive and motor‐cognitive training

**FIGURE 3:**
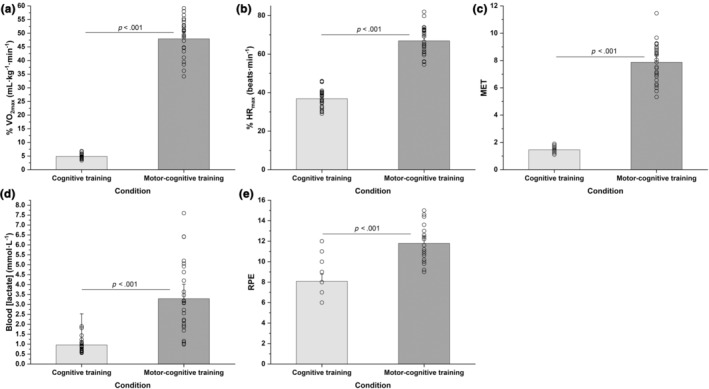
Average exercise intensity of computer‐based cognitive (Brain HQ) and motor‐cognitive (SKILLCOURT) sessions. (a) %VO_2max_; (b) %HR_max_; (c) metabolic equivalent (MET); (d) lactate; (e) rate of perceived exertion (RPE). Error bars reflect 95% confidence intervals. ***p < 0.001.

Significant differences between the computer‐based and motor‐cognitive (m‐c) session were observed for %VO_2max_ (*t*_25_ = 21.42, *p* < 0.001, d = 2.91), %HR_max_ (*t*_25_ = 20.87, *p* < 0.001, d = 4.09), MET levels (*t*_25_ = 21.7, *p* < 0.001, d = 4.29), blood [lactate] (*t*_25_ = 6.58, *p* < 0.001, d = 1.29), and RPE (*t*_*25*_ = 8.99, *p* < 0.001, d = 1.76). Differences between the cognitive and m‐c training session are illustrated in Figure 3.

### 3.2 Motor‐cognitive exercises

**FIGURE 4:**
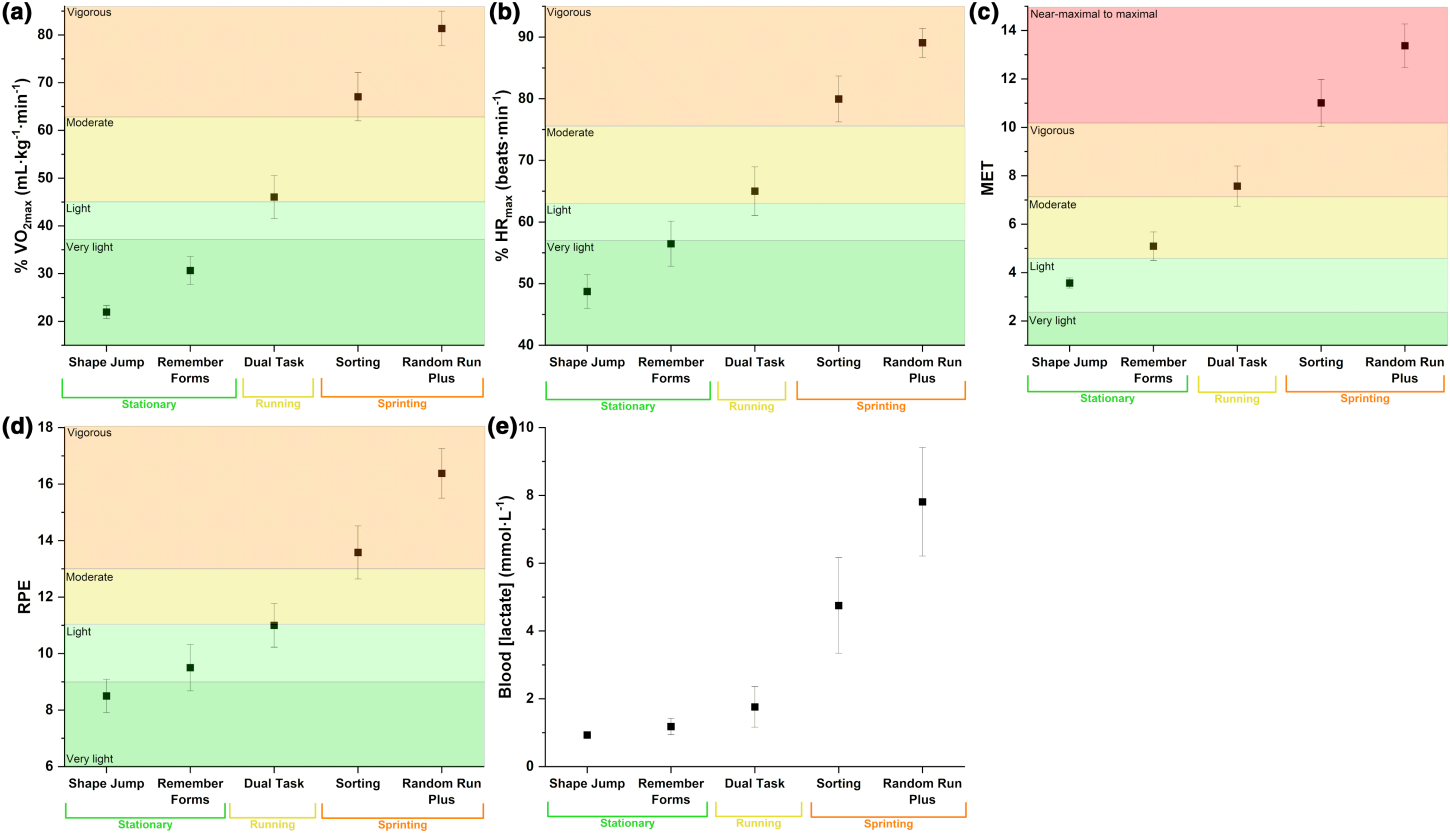
Results of the physiological demands of the motor‐cognitive SKILLCOURT exercises with the classification of exercise intensity range proposed by the ACSM (Garber et al., 2011). (a) % VO_2max_; (b) % HR_max_; (c) metabolic equivalent (MET); (d) rate of perceived exertion (RPE); (e) lactate. The classification of “stationary”, “running” and “sprinting” refers to the classification of motor tasks required for the different motor‐cognitive exercises (see methods section). Data points reflect mean ± 95% confidence interval.

Indicators of exercise intensity for all m‐c exercises on the SKILLCOURT are summarized in Table 1 and illustrated relative to the ACSM guidelines in Figure 4. Significant differences between m‐c tasks were observed for %VO_2max_ (*F*_(2.93,73.34)_ = 360.15, *p* < 0.001, *η*_*p*_^*2*^ = 0.94) and %HR_max_ (*F*_(2.76,69.07)_ = 282.43, *p* < 0.001, *η*_*p*_^*2*^ = 0.92). Post hoc tests revealed significant differences between all pairwise comparisons for both parameters (*p* < 0.001). The *Random Run Plus* was associated with the highest %VO_2max_ (81.35% ± 8.96) and %HR_max_ (89.08% ± 5.82) corresponding to vigorous intensity. The lowest intensity was observed for *Shape Jump* (21.96% (3.39%) VO_2max_; 48.73% ± (6.85) HR_max_) corresponding to very light intensity.

**TABLE 1:** Physiological responses to the motor‐cognitive exercises on the SKILLCOURT.

Exercise	% VO_2max_ (mL·kg^−1^·min^−1^)	% HR_max_ (b·min^−1^)	MET	RPE	Blood [lactate] (mmol·L^−1^)
Shape Jump	21.96 ± 3.39	48.73 ± 6.85	3.57 ± 0.51	8.5 ± 1.45	0.93 ± 0.24
Remember Forms	30.65 ± 7.28	56.46 ± 9.03	5.1 ± 1.47	9.5 ± 2.03	1.18 ± 0.60
Dual Task	46.04 ± 11.14	65 ± 9.77	7.57 ± 2.05	11 ± 1.9	1.76 ± 1.5
Sorting	67.04 ± 12.52	79.96 ± 9.25	11.01 ± 2.4	13.58 ± 2.32	4.75 ± 3.51
Random Run Plus	81.35 ± 8.96	89.08 ± 5.82	13.37 ± 2.09	16.38 ± 2.19	7.81 ± 3.95

*Note*: Values represent mean (±standard deviation).

METs were significantly different between motor‐cognitive exercises (*F*_(2.75,68.82)_ = 305.58, *p* < 0.001, *η*_*p*_^*2*^ = 0.93). Post hoc analyses showed significant differences between all pairwise comparisons (*p* < 0.001) indicating the lowest METs of 3.57 ± 0.5 in *Shape Jump* corresponding to light intensity and the highest METs of 13.37 ± 2.09 for the *Random Run Plus* corresponding to near‐maximal to maximal exercise intensity.

Significant differences were observed in RPE for m‐c tasks (*F*_(2.81,70.2)_ = 208.66, *p* < 0.001, *η*_*p*_^*2*^ = 0.89). Post hoc pairwise comparisons revealed significantly different RPEs between all exercises (*p* < 0.05) indicating the lowest RPE in *Shape Jump* (8.5 ± 1.45) corresponding to very light intensity and the highest RPE *Random Run Plus* (16.38 ± 2.19) corresponding to vigorous intensity.

Significant differences in blood [lactate] were shown between m‐c exercises (*F*_(1.38,34.51)_ = 63.76, *p* < 0.001, *η*_*p*_^*2*^ = 0.72). *Sorting* and *Random Run Plus* revealed significantly higher blood [lactate] when compared to all other tasks (*p* < 0.001). No significant differences were revealed between *Shape Jump*, *Remember Forms*, and *Dual Task* (*p* > 0.05). Blood [lactate] were the lowest after the *Shape Jump* (0.93 ± 0.24 mmol·L^−1^) and highest after the *Random Run Plus* (7.81 ± 3.95 mmol·L^−1^).

## 4 DISCUSSION

This is the first study which compares the physiological response of a computer‐based cognitive and a motor‐cognitive (m‐c) training. As hypothesized, the m‐c session provided a substantially higher physiological load when compared to the cognitive training alone. Further, by modulating the task design, the m‐c exercises on the SKILLCOURT technology covered the full range (very light to vigorous/near‐maximal) of exercise intensities. Importantly, considerably higher exercise intensities were reached when compared to many exergaming technologies. This makes m‐c training attractive for different target groups from patients to high level athletes to improve physical and cognitive performance and health. The immersive and attractive digital environment together with a variety of exercises may further allow variability of training, thereby increasing compliance, and support long‐term training adherence which is crucial for effective training on both the cardio‐vascular and neurophysiological level.

The m‐c training, including all exercises, was associated with a significantly higher physiological response when compared to the computer‐based cognitive training. This finding is consistent with previous studies demonstrating that sedentary conditions such as resting, watching TV, or playing video games, elicited lower physiological demands when compared to exergaming (Aygün & Çakır‐Atabek, 2023; Çakir‐Atabek et al., 2019; Graves et al., 2010; O'Donovan et al., 2012; White et al., 2011). It may be argued that for the computer‐based exercises in this study the higher cognitive engagement when compared to, for example, watching TV increases the VO_2_. In fact, Grassmann et al. (2016) reported an increased respiratory rate during cognitive tasks. Further, the blood supply to active cognitive regions increases during cognitively challenging tasks (Sorond et al., 2008). However, the effects of cognitive load on systemic VO_2_ are way less when compared to motor engagement (Backs & Seljos, 1994). Accordingly, VO_2_ is not an established parameter for determining cognitive load (Ayres et al., 2021). Our findings of significantly higher physiological demands as evidenced in all measured variables and throughout exercises during the m‐c training are therefore confirming the initial hypotheses that integrating even small motor tasks (stepping movement) into the cognitive exercise significantly increases VO_2_ when compared to computer‐based cognitive training.

According to the ACSM guidelines, the selected m‐c tasks covered all intensity ranges from very light/light (*Shape Jump*, *Remember Forms*), to moderate (dual‐task), and vigorous (*Sorting*, *Random Run Plus*) exercise intensities. The lowest intensity was observed for the “*Shape Jump*” which only requires stepping movements to the left and right. This resulted in an average of 21.9%VO_2max_ and an 48.7% HR_max_ which was still significantly higher when compared to the cognitive training (*p* < 0.001). These values are comparable to the results of Monedero et al. (2014) who found an 18.9%VO_2max_ intensity during a “Wii Sports” exergaming. Furthermore, Monedero et al. (2014) reported an energy expenditure of 3.3 MET, which is similar to the present study (3.57 MET). Current research recommends exercise intensities of about 60% of HR_max_ (Gonçalves et al., 2021) in patients with cardiovascular diseases. Therefore m‐c exercises like shape‐jump (48.7% HR_max_) and Remember Forms (56.5% HR_max_) can be suggested as suitable to enhance cardiovascular capabilities in this population. The structure of the m‐c exercises is intermittent where the exercise is typically performed for 1 min. This provides flexibility to design, for example, a 10 × 1 training protocol (1‐min exercise, 1‐min rest), which is one of the most frequently used and most effective intermittent training protocols for patient populations but also for elderly in general (Marriott et al., 2021).

In addition to cardiac disease patients, m‐c training with small movement amplitudes and complexity (i.e., stepping movements) may be promising for people with limitations in locomotor abilities such as Parkinsons disease (Bode et al., 2023). Importantly, in contrast to computer‐based cognitive training, the cardiovascular load is still significantly higher despite limited movement dynamics and complexity. As such, patients and elderly people may not only benefit from the cognitive but also cardiovascular training effects (Johansson et al., 2023). Even short bouts of moderate exercise around 5–10 min three times a day at 50%–70% HR_max_ have been shown sufficient to improve cardiovascular function in the sedentary elderly population (Magutah et al., 2020).

The game‐like character of the m‐c exercises further supports motivation, enjoyment (Moholdt et al., 2017; Monedero et al., 2014), and long‐term training adherence (Kramer et al., 2014). This has previously been identified as a crucial factor for long‐term training success also for the elderly population (Brouillette et al., 2023). As exergaming has the potential to reach higher adherence rates when compared to, for example, aerobic training (Karssemeijer et al., 2019), participants may not only benefit from cognitive and cardiovascular training stimuli but also long‐term training motivation.

The highest exercise intensity was reached for the *Random Run Plus* training. With an average intensity of 81.4% VO_2max_ and 89.1% HR_max_, this m‐c exercise can be considered as vigorous (Garber et al., 2011) and in the same intensity range as previous high‐intensity training in running or cycling (Pratt et al., 2013; Vaccari et al., 2020). 13.7 MET's, an RPE of >16 and 7.8 mmol/L in blood [lactate] following the *Random Run Plus* support the high‐intensity nature of this type of motor‐cognitive exercise. Importantly, the reached %VO_2max_ and HR_max_ values for *Random Run Plus* were approximately 15%–20% higher when compared to other exergames, such as the ExerCube (Ketelhut et al., 2021) or cognitive tasks performed concomitant with cycling an ergometer (Berg & Moholdt, 2020). The same applied also to MET, RPE, and [blood lactate] values which substantially exceeded previous exergames (Aygün & Çakir‐Atabek, 2021; Aygün & Çakır‐Atabek, 2023; Barry et al., 2016; Perusek et al., 2014; Röglin et al., 2021). These findings suggest that despite motor‐cognitive interference and dual‐task costs that are expected in motor‐cognitive exercises and may limit the maximum exercise intensity, physiological demands corresponding to high‐intensity training can still be reached.

The high exercise intensity observed in the current study can be explained by the highly dynamic, whole‐body movements which require reactive agility and changes of direction. On the 4 m × 4 m court, participants had to cover greater distances when compared to most exergames, where exercises are often limited to either upper or lower extremity and a lower with less full‐body and reactive agility movements (Çakir‐Atabek et al., 2019; Ketelhut et al., 2021; O'Donovan et al., 2012; Perusek et al., 2014; Worley et al., 2011). Motor‐cognitive (m‐c) training on the SKILLCOURT thus also qualifies for short‐term high‐intensity exercise training which is promising for trained athletes (Engel et al., 2018; Liu et al., 2024) but also beneficial for the elderly population (Marriott et al., 2021; Rebsamen et al., 2019).

Motor‐cognitive training is not only designed for cardiovascular adaptations, but it should support the improvement of cognitive abilities. As such, each exercise performed in this study addressed specific cognitive abilities. To induce neural plasticity, physical exercise is a key component as neurotrophic factors such as BDNF are released and support synapse formation and neuron survival (Knaepen et al., 2010; Wang et al., 2022). Reycraft et al. (2020) recently compared the effects of different exercise protocols (30‐min moderate intensity running, 30‐min vigorous intensity running, 4 × 30 s all‐out running) on blood plasma BDNF concentration. It was observed that BDNF only increased in vigorous and all‐out exercise conditions. Further, the all‐out intermittent protocol revealed higher BDNF concentrations following exercise when compared to both the moderate and vigorous continuous exercise. A correlation analysis demonstrated a direct positive relation between exercise‐induced changes in blood [lactate] and changes in BDNF concentration further supporting the direct relation between exercise intensity and neurotrophic factor release. Together, this emphasizes the potential of high‐intensity intermittent motor‐cognitive exercises to support the release of neurotrophic factors. As the *Random Run Plus* but also the *Sorting* exercises are designed as intermittent all‐out exercises, these tasks may provide a strong stimulation for BDNF release and thus follow the guided plasticity facilitation model (Herold et al., 2018). As the combination of physical and cognitive exercise has previously been shown to be more effective than computer‐based training (Tait et al., 2017), higher BDNF levels may be a possible contributing factor.

## 5 LIMITATIONS AND FUTURE DIRECTION

While this study was focusing on the cardiovascular response and intensity profile of different m‐c training tasks, it did not measure the release of BDNF. We can therefore only speculate about the effects on neurotrophic factors associated with different intensities of m‐c exercises. Future research needs to address the response of neurotrophic factors during motor‐cognitive training to further elaborate on the physiological foundation of motor‐cognitive training effects in line with the guided plasticity facilitation hypothesis (Herold et al., 2018). Further, we used a 4 m × 4 m court for the study. As the technology is also available with 3 m × 3 m and 5 m × 5 m courts, it needs to be investigated how the physiological profile of m‐c training does change with different court sizes. Further research should also elaborate on the transferability of results to different target populations including patients or elderly people.

## 6 CONCLUSION

The results suggest that the applied m‐c exercises on the SKILLCOURT address the full range of exercise intensities. Especially for high‐intensity exercise, m‐c tasks exceed the physical load previously reported for exergaming. As such, m‐c exercises might qualify for endurance training in different target groups ranging from cardiac disease patients to high level athletes. The immersive and enjoyable training design may support participant engagement and increase adherence which is essential for training success. Further, m‐c tasks may not only support cardiovascular adaptations but also cognitive performance gains through the release of neurotrophic factors.

## FUNDING INFORMATION

This research project did not receive financial support.

## CONFLICT OF INTEREST STATEMENT

TH provides scientific consultancy to SKILLCOURT GmbH. There is no financial or other interest in the product or distributor of the product. The company was not involved in any aspect of the research project.

## ETHICS STATEMENT

The study was approved by the Luxembourgish national research ethics committee (CNER) in accordance with the Declaration of Helsinki (202207/01 v2.0).

## Data Availability

The data will not be made publicly available due to ethical reasons. For researchers who meet the criteria for access to confidential data, the data will be made available on request.
